# Supplementary material for: Resolving the full spectrum of human genome variation using Linked-Reads
Source: Genome Res. 2019 Apr;29(4):635–45. doi: 10.1101/gr.234443.118 (PMC6442396; doi:10.1101/gr.234443.118)
Supplement: Supplemental Material [file supp_gr.234443.118_Supplemental_longranger-2.2.zip › longranger-2.2.tar.bz2!/lr2.2-public/lib/stan/cmdstan/stan/doc/api/html/integrate__ode_8hpp.html]

Stan: src/stan/math/ode/integrate\_ode.hpp File Reference


|  |  |
| --- | --- |
|  | Stan  2.5.0  probability, sampling & optimization |


- Main Page
- Related Pages
- Namespaces
- Classes
- Files

- File List
- File Members

All Classes Namespaces Files Functions Variables Typedefs Enumerations Enumerator Friends Macros Pages

- src
- stan
- math
- ode

Namespaces |
Functions

integrate\_ode.hpp File Reference

`#include <ostream>`  
`#include <vector>`  
`#include <boost/numeric/odeint.hpp>`  
`#include <stan/meta/traits.hpp>`  
`#include <stan/math/functions/value_of.hpp>`  
`#include <stan/math/error_handling/check_less.hpp>`  
`#include <stan/math/error_handling/check_finite.hpp>`  
`#include <stan/math/error_handling/matrix/check_nonzero_size.hpp>`  
`#include <stan/math/error_handling/matrix/check_ordered.hpp>`  
`#include <stan/math/ode/coupled_ode_system.hpp>`  
`#include <stan/math/ode/coupled_ode_observer.hpp>`

Go to the source code of this file.

|  |  |
| --- | --- |
| Namespaces | |
|  | stan |
|  | Probability, optimization and sampling library. |
|  | |
|  | stan::math |
|  | Matrices and templated mathematical functions. |
|  | |

|  |  |
| --- | --- |
| Functions | |
| template<typename F , typename T1 , typename T2 > | |
| std::vector< std::vector  < typename stan::return\_type  < T1, T2 >::type > > | stan::math::integrate\_ode (const F &f, const std::vector< T1 > y0, const double t0, const std::vector< double > &ts, const std::vector< T2 > &theta, const std::vector< double > &x, const std::vector< int > &x\_int, std::ostream \*msgs) |
|  | Return the solutions for the specified system of ordinary differential equations given the specified initial state, initial times, times of desired solution, and parameters and data, writing error and warning messages to the specified stream. More... |
|  | |


---

[ Stan Home Page ]

*© 2011–2014,
Stan Development Team.*
